# Health-related quality of life of carfilzomib- and daratumumab-based therapies in patients with relapsed/refractory multiple myeloma, based on German benefit assessment data

**DOI:** 10.1007/s11136-019-02307-5

**Published:** 2019-09-24

**Authors:** Katja Weisel, Heinz Ludwig, Achim Rieth, Andrea Lebioda, Hartmut Goldschmidt

**Affiliations:** 1 Medizinische Klinik und Poliklinik (Onkologie, Hämatologie, Knochenmarktransplantation mit Abteilung für Pneumologie), Universitätsklinikum Hamburg – Eppendorf, II., Martinistr. 52, 20246 Hamburg, Germany; 2 Department of Medicine II, University Hospital Tubingen, Tübingen, Germany; 3 1st Department of Medicine, Wilhelminen Cancer Research Institute, Vienna, Austria; 4 Amgen GmbH, Munich, Germany; 5 Internal Medicine V and National Center for Tumor Diseases (NCT), University Clinic Heidelberg, Heidelberg, Germany

**Keywords:** Quality of life, Daratumumab, Carfilzomib, Multiple myeloma, Indirect comparison

## Abstract

**Background:**

Carfilzomib and daratumumab are licensed in relapsed/refractory multiple myeloma (RRMM), but no head-to-head trials have been conducted.

**Methods:**

We used data from dossiers prepared for the German Federal Joint Committee based on two phase III randomized trials of carfilzomib-based therapies (ASPIRE, ENDEAVOR) and two of daratumumab-based therapies (POLLUX, CASTOR) to conduct a descriptive assessment of health-related quality of life (HRQoL). HRQoL was assessed using the European Organisation for Research and Treatment of Cancer 30-item HRQoL Questionnaire, with hazard ratios calculated for carfilzomib- and daratumumab-based therapy versus comparators for time to HRQoL deterioration of ≥ 10 points. Analyses were also conducted on data from the EORTC 20-item myeloma-specific questionnaire, the Functional Assessment of Cancer Therapy/Gynecologic Oncology Group-Neurotoxicity scale, and the visual analog scale of the EuroQoL 5-dimension, 5-level questionnaire, where results for these instruments were available. As the designs and patient population of the four trials were similar but not identical, the analysis included only indirect, descriptive comparisons.

**Results:**

Compared with lenalidomide/dexamethasone, median time to deterioration in global health status/QoL was longer for carfilzomib-based therapy versus control, but similar for daratumumab-based therapy and control. Compared with bortezomib/dexamethasone, time to deterioration was significantly longer for carfilzomib-based therapy versus control for global health status/QoL and numerous functional and symptom subscales. HRQoL measurement is feasible in large RRMM populations.

**Conclusion:**

Descriptive assessment of HRQoL data suggests potential benefits for carfilzomib-based over daratumumab-based therapy.

**Electronic supplementary material:**

The online version of this article (10.1007/s11136-019-02307-5) contains supplementary material, which is available to authorized users.

## Introduction

Multiple myeloma (MM) is an incurable, relapsing disease that is generally diagnosed in elderly individuals; median age at diagnosis is 72 years for men and 74 years for women [1–3]. In recent years, mortality rates in MM have improved significantly as a result of advances in treatment [4]. The aim of treatment has therefore shifted from purely palliative therapy to the early use of potent treatments to prolong disease control and improve overall survival (OS) [5–8].

The World Health Organization defines quality of life (QoL) as “an individual’s perception of their position in life in the context of the culture and value systems in which they live and in relation to their goals, expectations, standards and concerns” [9]. Health-related QoL (HRQoL) can be additionally defined as the functional effect of a medical condition and its consequent therapy upon a patient [10]. Patients with MM often experience substantial adverse effects on HRQoL, including pain, fatigue, and breathlessness, as well as impaired physical functioning [11–13]. Furthermore, treatment-related toxicity can also negatively affect patients’ HRQoL [14]. During treatment, HRQoL is generally maintained at baseline levels or declines, although improvements in some HRQoL domains may be seen [15–19]. As a result, QoL has become an important determinant of therapy, with some physicians and patients choosing to optimize QoL at the cost of prolonged survival [20]. QoL is also a central component of health technology and cost-effectiveness assessment [21]. Incorporation of QoL endpoints in clinical trials is therefore essential to allow better clinical decision-making in patients with MM [22], including those with relapsed/refractory (RR) MM.

Carfilzomib is an epoxyketone proteasome inhibitor that binds selectively and irreversibly to the constitutive proteasome and immunoproteasome. In the phase III ASPIRE and ENDEAVOR trials, carfilzomib-based therapy was associated with significantly prolonged progression-free survival (PFS) and OS compared with controls [5, 8, 23, 24]. Based on these two studies, carfilzomib is licensed in Europe and the USA in combination with either lenalidomide and dexamethasone or dexamethasone alone for the treatment of adults with RRMM who have received at least one prior therapy [25, 26]. Furthermore, the European Summary of Product Characteristics notes the benefits of carfilzomib on HRQoL as reported in the ASPIRE and ENDEAVOR studies. In the USA, carfilzomib is also licensed as monotherapy in patients with RRMM who have received one or more lines of therapy [26].

Daratumumab is a monoclonal antibody specific for CD38, which is overexpressed in hematological malignancies such as MM [27]. The phase III CASTOR and POLLUX trials of combinations including daratumumab showed significantly prolonged PFS versus controls [28, 29]. As a result, daratumumab is licensed in Europe as monotherapy for the treatment of adults with RRMM whose prior therapy included a proteasome inhibitor and an immunomodulatory agent and who have progressed while receiving their most recent therapy. It is also licensed in combination with lenalidomide/dexamethasone or bortezomib/dexamethasone for the treatment of adults with MM who have received at least one prior therapy [30]. In the USA, daratumumab is licensed: (1) in combination with bortezomib, melphalan and prednisone for the treatment of patients with newly diagnosed MM who are ineligible for autologous stem cell transplant; (2) in combination with lenalidomide and dexamethasone, or bortezomib and dexamethasone, for the treatment of patients with MM who have received at least one prior therapy; (3) in combination with pomalidomide and dexamethasone for the treatment of patients with MM who have received at least two prior therapies including lenalidomide and a proteasome inhibitor; and (4) as monotherapy for the treatment of patients with MM who have received at least three prior lines of therapy including a proteasome inhibitor and an immunomodulatory agent or who are double-refractory to both of these drug classes [31].

To date, there have been no direct comparisons between carfilzomib and daratumumab to permit an assessment of their relative impacts on HRQoL, or efficacy and tolerability endpoints. However, both carfilzomib and daratumumab have recently gone through the German Federal Joint Committee (Gemeinsamer Bundesausschuss; G-BA) early benefit assessment process according to §35a social code book V (under Arzneimittelmarkt-Neuordnungsgesetz [AMNOG; ‘Pharmaceuticals Market Reorganisation Act’]) and the respective dossiers prepared by the pharmaceutical companies are publicly available. Here, we used these public-domain dossier data to conduct a descriptive, indirect comparison of carfilzomib and daratumumab and their effects on HRQoL as reported from the ASPIRE, ENDEAVOR, CASTOR, and POLLUX trials [32, 33].

## Methods

### Trial designs

All four trials were phase III, randomized, open-label trials including patients with RRMM [5, 8, 23, 24, 28, 29, 32–37] (Table 1). All four trials were conducted in accordance with the Declaration of Helsinki and all participants provided written, informed consent. The primary endpoint in each trial was PFS, with OS and HRQoL assessed prospectively as secondary endpoints.

**Table 1:** Summary of the trials included in the analysis

	Carfilzomib-based therapy				Daratumumab-based therapy			
	ASPIRE		ENDEAVOR		POLLUX		CASTOR	
Trial design	Randomized (1:1), controlled, open-label, multicenter, phase III trial Treatment until progression, unacceptable toxicity, or withdrawal of consent^a^							
Treatment arms	KRd vs. Rd		Kd vs. Vd		DRd vs. Rd		DVd vs. Vd	
Enrolment	*N* = 792 (KRd *n* = 396; Rd *n* = 396) 129 centers in 20 countries		*N* = 929 (Kd *n* = 464; Vd *n* = 465) 198 centers in 27 countries		*N* = 569 (DRd *n* = 286; Rd *n* = 283) 136 centers in 18 countries		*N* = 498 (DVd *n* = 251; Vd *n* = 247) 115 centers in 16 countries	
Stratification	β2-microglobulin level, prior lenalidomide and bortezomib		Prior PI therapy, number of prior therapies, ISS stage, route of bortezomib administration (sc/iv)		ISS stage, number of prior therapies, prior lenalidomide		ISS stage, number of prior therapies, prior bortezomib	
Patient population	1–3 prior lines of therapy (median, 2) ECOG status 0–2 Median age: 64 years Male: KRd 54%; Kd 58%		1–3 prior lines of therapy (1 prior therapy: 50%) ECOG status 0–2 Median age: 65 years Male: Kd 52%; Vd 49%		≥ 1 prior line of therapy (median, 1) Median age: 65 years Male: DRd 60%; Rd 58%		≥ 1 prior line of therapy (median: 2) Median age: 64 years Male: DVd 55%; Vd 60%	
Quality of life assessment	Day 1 of cycles 3, 6, 12 and 18 End of treatment (30 days after last dose)		Day 1 of every cycle until disease progression, discontinuation, or subsequent therapy		Day 1 of every cycle and after end of treatment in weeks 4, 8 and 16		Day 1 of every cycle and after end of treatment on week 4, with disease progression on weeks 8 and 16	
Overall survival^b^	KRd (*n* = 396)	Rd (*n* = 396)	Kd (*n* = 464)	Vd (*n* = 465)	DRd (*n* = 286)	Rd (*n* = 283)	Vd (*n* = 247)	DVd (*n* = 251)
Median (95% CI) (months)	48.3 (42.4, 52.8)	40.4 (33.6, 44.4)	47.6 (42.5, –)	40.0 (32.6, 42.3)	– (–)	– (–)	– (–)	– (–)
Hazard ratio (95% CI)	0.79 (0.67, 0.95)		0.79 (0.65, 0.96)		0.63 (0.42, 0.95)		0.63 (0.42, 0.96)	
*p* value	0.009		0.020		0.027		0.029	

^a^Maximum of eight cycles Vd cycles in CASTOR, followed by daratumumab monotherapy in the DVd arm; maximum of 18 cycles of KRd in ASPIRE, followed by Rd

^b^Overall survival reported as presented in the Arzneimittelmarkt-Neuordnungsgesetz dossiers, and previously published for carfilzomib and presented at congresses for daratumumab [5, 8, 23, 24, 28, 29, 32–37]

*CI* confidence interval, *DRd* daratumumab/lenalidomide/dexamethasone, *DVd* daratumumab/bortezomib/dexamethasone, *ECOG* Eastern Cooperative Oncology Group, *ISS* International Staging System, *iv* intravenous, *Kd* carfilzomib/dexamethasone, *KRd* carfilzomib/lenalidomide/dexamethasone, *PI* proteasome inhibitor, *Rd* lenalidomide/dexamethasone, *sc* subcutaneous, *Vd* bortezomib/dexamethasone

The efficacy and safety of carfilzomib-based therapy were evaluated in the ASPIRE and ENDEAVOR studies. In ASPIRE (ClinicalTrials.gov: NCT01080391; EudraCT: 2009-016839-35), patients received lenalidomide (25 mg) and dexamethasone (40 mg), with or without carfilzomib (20 mg/m^2^ on days 1 and 2 of cycle 1; 27 mg/m^2^ thereafter twice weekly with the frequency reduced to once every 2 weeks after 12 cycles) [24]. In ENDEAVOR (ClinicalTrials.gov: NCT01568866; EudraCT: 2012-000128-16), patients received dexamethasone (20 mg) with either carfilzomib (20 mg/m^2^ on days 1 and 2 of cycle 1; 56 mg/m^2^ thereafter) or bortezomib (1.3 mg/m^2^) [23].

Daratumumab-based treatment was evaluated in the POLLUX and CASTOR studies. In POLLUX (ClinicalTrials.gov: NCT02076009; EudraCT: 2013-005525-23), patients received lenalidomide (25 mg) and dexamethasone (40 mg), with or without daratumumab (16 mg/kg given weekly for 8 weeks, followed by dosing every 2 weeks for 16 weeks, and every 4 weeks thereafter) [28], while in CASTOR (ClinicalTrials.gov: NCT02136134; EudraCT: 2014-000255-85), patients received dexamethasone (20 mg) and bortezomib (1.3 mg/m^2^), with or without daratumumab (16 mg/kg given weekly for 9 weeks, every 3 weeks for 15 weeks, and every 4 weeks thereafter) [29].

Treatment generally continued until disease progression, unacceptable toxicity, or withdrawal of consent. However, in CASTOR, a maximum of eight cycles of bortezomib and dexamethasone was permitted. Similarly, in ASPIRE, only 18 cycles of carfilzomib, lenalidomide, and dexamethasone were permitted, followed by lenalidomide plus dexamethasone [23, 24, 28, 29].

### Quality of life assessment

The European Organisation for Research and Treatment of Cancer (EORTC) 30-item QoL Questionnaire (QLQ-C30) was used in all four trials to assess HRQoL [32, 33, 35, 36]. This questionnaire, which includes both specific functional and symptom subscales as well as an assessment of global health status, has been extensively validated and is widely used for assessment of HRQoL in patients with cancer [38]. In ASPIRE and ENDEAVOR, HRQoL was also assessed using the EORTC 20-item myeloma-specific questionnaire (QLQ-MY20) and, in ENDEAVOR only, the Functional Assessment of Cancer Therapy/Gynecologic Oncology Group-Neurotoxicity scale (FACT/GOG-Ntx) was also used. In addition, HRQoL was assessed in CASTOR and POLLUX using the visual analog scale of the EuroQoL 5-dimension, 5-level questionnaire (EQ-5D-VAS). HRQoL was assessed on day 1 of some or all cycles, as well as at other pre-planned timepoints, depending on the trial (Table 1).

### Data synthesis and analysis

All reported data are derived from public-domain dossiers, as part of the AMNOG assessment by G-BA. Adherence to HRQoL assessment was recorded throughout the studies and return rates calculated for each questionnaire, based on the number of patients alive and receiving study treatment for each trial. Hazard ratios (HRs) were calculated for carfilzomib- and daratumumab-based therapy versus comparators for time to HRQoL deterioration of ≥ 10 points on the EORTC QLQ-C30. For carfilzomib, 95% confidence intervals (CIs) were also calculated. In ASPIRE and ENDEAVOR, time to a ≥ 10-point deterioration was also assessed on the EORTC QLQ-MY20. Time to a ≥ 5-point deterioration on the FACT/GOG-Ntx was evaluated for ENDEAVOR, and time to a ≥ 7-point deterioration on the EQ-5D-VAS assessed in POLLUX and CASTOR. Calculations were based on intention-to-treat (ITT) populations. The minimal important difference (MID) for the EORTC QLQ-C30 has been reported as 8–12 points in patients with MM [39], and an MID of 10 points is recommended for identifying clinically relevant differences in worsening of HRQoL [40]. Furthermore, patients with an increase of ≥ 10 points generally report a subjective improvement of ‘moderate’ or better [39, 41, 42].

For completeness, summary data for PFS and OS from these studies are included, as have been reported previously [5, 8, 34, 37]. In brief, PFS and OS were analyzed by the Kaplan–Meier method, and median PFS and OS were calculated with 95% CIs for study treatment and comparator. HRs and 95% CIs were also calculated for time to first adverse event, serious adverse event, severe adverse event (Grade ≥ 3), discontinuation from any study medication, and discontinuation from all study medications. For the ENDEAVOR head-to-head comparison, relative risks and 95% CIs for time to occurrence of peripheral neuropathy symptoms Grade ≥ 2 and Grade ≥ 3 were also calculated.

As the designs and patient population of the four trials were similar but not identical (Table 1), the present analysis includes only indirect, descriptive comparisons between carfilzomib- and daratumumab-based therapy.

## Results

### Patient population and return rates for quality of life questionnaires

In total, 2788 patients across the four studies were included in the analyses. Patient numbers in the ITT population for each treatment arm in the four studies are shown in Table 1. Return rates for HRQoL questionnaires were > 84% at baseline and remained high throughout treatment in all four trials (Supplemental Table 1).

### Quality of life: carfilzomib-based therapy versus lenalidomide/dexamethasone

**Fig. 1:**
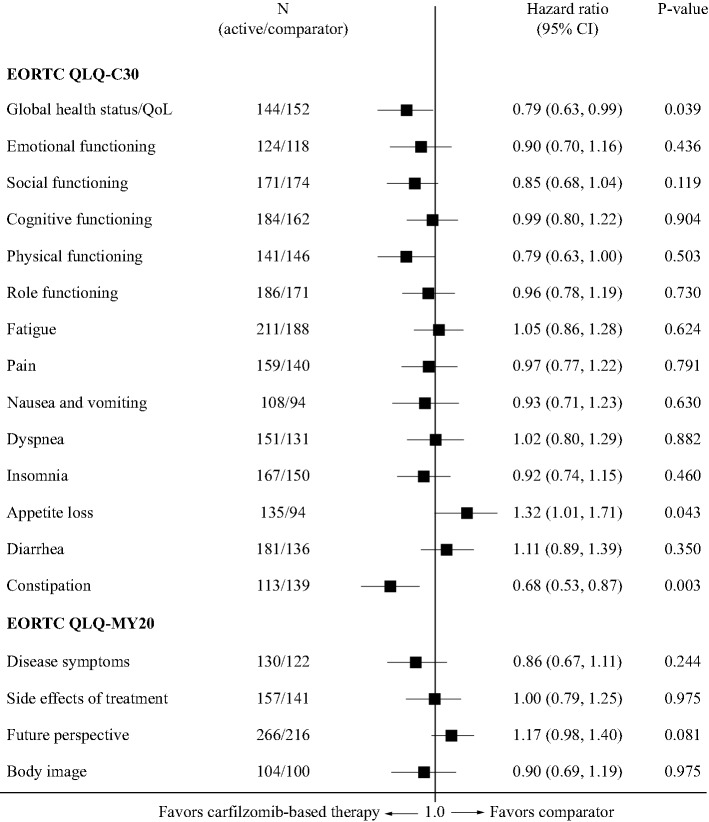
Forest plot showing hazard ratios for differences between lenalidomide/dexamethasone and carfilzomib/lenalidomide/dexamethasone (ASPIRE trial) for time to ≥ 10-point deterioration on the EORTC QLQ-C30 and in subscales of the EORTC QLQ-MY20. *CI* confidence interval, *EORTC* European Organisation for Research and Treatment of Cancer, *QLQ*-*C30* EORTC 30-item Quality of Life Questionnaire, *QLQ*-*MY20* EORTC 20-item myeloma-specific questionnaire, *QoL* quality of life

Median time to ≥ 10-point deterioration on the EORTC QLQ-C30 subscales is shown in Table 2. In ASPIRE, median time (in months) to deterioration in global health status/QoL was consistently longer for carfilzomib-based therapy versus control. Results on the global health status/QoL, physical functioning, and constipation subscales significantly favored carfilzomib-based therapy, while appetite loss favored lenalidomide/dexamethasone (Fig. 1; Table 2). There were no significant differences between treatment arms in any of the other subscales of the EORTC QLQ-C30, nor on the EORTC QLQ-MY20 (Fig. 1).

**Table 2:** Time to ≥ 10-point deterioration on the EORTC QLQ-C30 and subscales with carfilzomib-related therapy (ASPIRE and ENDEAVOR) and daratumumab-based therapy (POLLUX and CASTOR) versus comparator

	ASPIRE^a^		ENDEAVOR^a^		POLLUX^b^		CASTOR^b^	
	KRd (*n* = 396)	Rd (*n* = 396)	Kd (*n* = 464)	Vd (*n* = 465)	DRd (*n* = 286)	Rd (*n* = 283)	DVd (*n* = 251)	Vd (*n* = 247)
Functional scales								
Global health status/QoL	**16.6 (15.9, –)**	**11.9 (10.3, –)**	**3.8 (2.9, 4.7)**	**2.8 (2.8, 3.5)**	4.7	4.7	3.5	3.7
Emotional functioning	18.5 (16.4, –)	– (16.2, –)	7.0 (5.6, 11.2)	6.4 (4.7, 7.5)	6.6	7.8	5.7	4.4
Social functioning	15.9 (10.3, 16.6)	10.3 (4.9, 15.9)	**2.8 (2.8, 3.8)**	**2.8 (2.8, 3.7)**	**3.8**	**2.9**	3.0	3.0
Cognitive functioning	11.3 (10.3, 15.9)	10.5 (6.1, 15.9)	**4.7 (3.8, 6.6)**	**3.8 (2.9, 4.9)**	4.9	4.6	3.5	3.4
Physical functioning	**10.3 (5.2, 15.9)**	**10.3 (5.7, 15.8)**	**5.6 (4.7, 7.5)**	**3.8 (3.3, 5.6)**	5.9	7.5	4.3	4.2
Role functioning	17.1 (16.4, 21.3)	15.9 (10.5, 16.4)	2.8 (1.9, 2.9)	2.8 (2.2, 3.3)	3.7	3.1	2.3	2.8
Symptom scales								
Fatigue	4.7 (4.7, 10.3)	5.7 (4.7, 10.5)	1.9 (1.9, 2.0)	1.9 (1.8, 2.6)	1.9	2.0	1.6	2.1
Pain	16.1 (10.9, 17.0)	16.0 (11.0, –)	5.6 (4.7, 7.1)	4.0 (3.5, 5.6)	5.6	5.6	3.5	3.7
Nausea/vomiting	21.3 (16.5, 21.3)	17.2 (17.2, –)	**17.9 (11.2, –)**	**8.4 (6.6, 12.0)**	13.9	10.3	7.3	–
Dyspnea	16.4 (15.9, –)	17.3 (15.0, –)	2.9 (2.8, 3.8)	3.8 (2.9, 4.9)	5.5	5.7	3.5	2.9
Insomnia	15.9 (10.3, 16.3)	15.9 (10.3, 16.2)	**3.7 (2.8, 4.7)**	**2.8 (1.9, 3.5)**	6.6	3.7	2.4	2.9
Appetite loss	**16.5 (16.1, –)**	**– (16.4, –)**	**11.2 (9.4, –)**	**5.5 (4.6, 6.9)**	7.2	10.2	5.0	5.9
Diarrhea	15.9 (10.5, 15.9)	15.9 (10.8, 16.3)	**10.3 (8.4, 15.1)**	**5.6 (4.7, 7.5)**	5.6	5.7	5.7	6.9
Constipation	**17.5 (16.6, –)**	**16.1 (10.6, –)**	**– (15.2, –)**	**4.7 (3.6, 7.3)**	4.7	3.3	–	7.3

^a^Data are presented as median (95% confidence interval) months (calculated as days/30)

^b^Data are presented as median months. No 95% confidence intervals were presented for daratumumab data in the dossiers

**Fig. 2:**
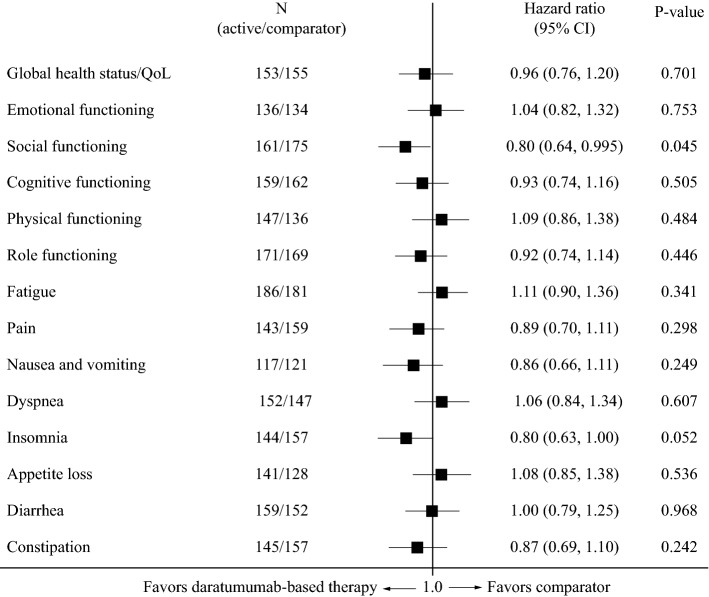
Forest plot showing hazard ratios for differences between lenalidomide/dexamethasone and daratumumab/lenalidomide/dexamethasone (POLLUX trial) for time to ≥ 10-point deterioration on the EORTC QLQ-C30. *CI* confidence interval, *EORTC* European Organisation for Research and Treatment of Cancer, *QLQ*-*C30* EORTC 30-item Quality of Life Questionnaire, *QoL* quality of life

**Fig. 3:**
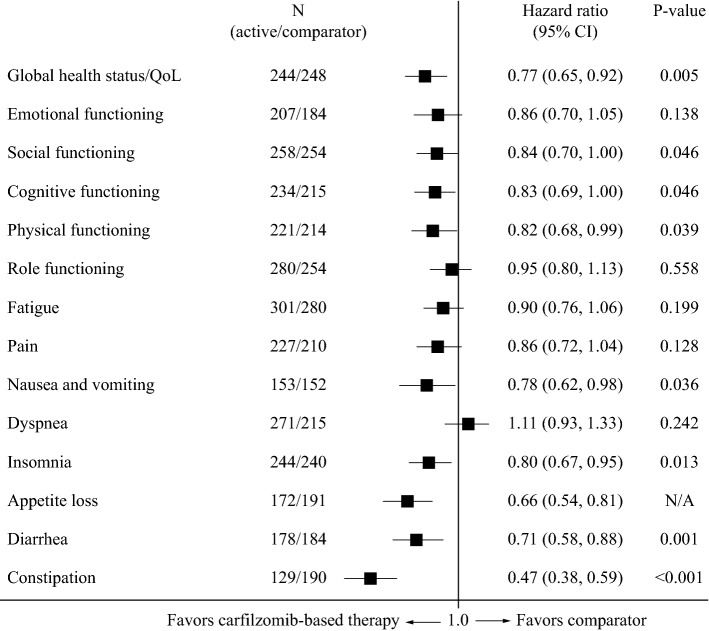
Forest plot showing hazard ratios for differences between bortezomib/dexamethasone and carfilzomib-based therapy (ENDEAVOR trial) for time to ≥ 10-point deterioration in subscales of the EORTC QLQ-C30. *CI* confidence interval, *EORTC* European Organisation for Research and Treatment of Cancer, *QLQ*-*C30* EORTC 30-item Quality of Life Questionnaire, *QoL* quality of life

**Fig. 4:**
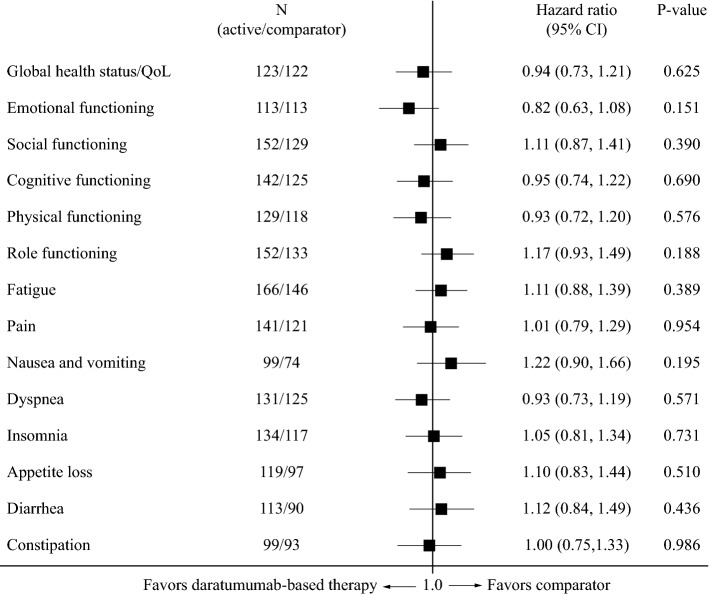
Forest plot showing hazard ratios for differences between bortezomib/dexamethasone and daratumumab-based therapy (CASTOR trial) for time to ≥ 10-point deterioration in subscales of the EORTC QLQ-C30. *CI* confidence interval, *EORTC* European Organisation for Research and Treatment of Cancer, *QLQ*-*C30* EORTC 30-item Quality of Life Questionnaire, *QoL* quality of life

Statistically significant results (see Figs. 1, 2, 3 and 4) are highlighted in bold

*DRd* daratumumab/lenalidomide/dexamethasone, *DVd* daratumumab/bortezomib/dexamethasone, *EORTC* European Organisation for Research and Treatment of Cancer, *Kd* carfilzomib/dexamethasone, *KRd* carfilzomib/lenalidomide/dexamethasone, *QoL* quality of life, *QLQ*-*C30* EORTC 30-item Quality of Life Questionnaire, *Rd* lenalidomide/dexamethasone, *Vd* bortezomib/dexamethasone

### Quality of life: daratumumab-based therapy versus lenalidomide/dexamethasone

Median time to ≥ 10-point deterioration on the EORTC QLQ-C30 subscales is shown in Table 2. In POLLUX, median time to deterioration in global health status/QoL did not differ between daratumumab-based therapy and control. Results on the social functioning subscale significantly favored daratumumab-based therapy, with no significant differences between treatment arms on any other subscale (Fig. 2; Table 2). In addition, there were no significant differences between treatment arms on the EQ-5D-VAS (HR 0.97; 95% CI 0.78, 1.21).

### Quality of life: carfilzomib-based therapy versus bortezomib/dexamethasone

Median times to ≥ 10-point deterioration in the EORTC QLQ-C30 subscales are shown in Table 2. In ENDEAVOR there were significant differences favoring carfilzomib-based therapy in global health status/QoL and on numerous functional (social, cognitive and physical) and symptom (insomnia, appetite loss, nausea and vomiting, diarrhea, and constipation) subscales of the EORTC QLQ-C30 (Fig. 3; Table 2). There were no significant differences between treatment arms in the remaining subscales. In this trial, treatment continued until disease progression (a median of eight cycles bortezomib/dexamethasone was given) (Table 1). On the EORTC QLQ-MY20, there was a significant difference in favor of carfilzomib-based therapy on the side effects subscale, but not the disease symptoms, future perspective, or body image subscales (Supplemental Fig. 1), and on the FACT/GOG-NTx, there was a significant difference in favor of carfilzomib-based therapy versus the comparator arm (HR 0.84; 95% CI 0.40, 1.28; *p* < 0.001).

### Quality of life: daratumumab-based therapy versus bortezomib/dexamethasone

Median times to ≥ 10-point deterioration in the EORTC QLQ-C30 subscales are shown in Table 2. In CASTOR there were no significant differences on any subscales of the EORTC QLQ-C30 between daratumumab-based therapy and control (Fig. 4; Table 2), and no significant difference on the EQ-5D-VAS (HR 1.00; 95% CI 0.79, 1.28).

### Overall and progression-free survival and adverse events

All four trials reported significantly improved HRs for OS for study treatment (carfilzomib- or daratumumab-based therapy) versus control (Table 1). In ASPIRE, median OS was 48.3 months with carfilzomib/lenalidomide/dexamethasone versus 40.4 months with lenalidomide/dexamethasone [8], and in ENDEAVOR, median OS was 47.6 months with carfilzomib/dexamethasone versus 40.0 months with bortezomib/dexamethasone [5] (Table 1). In POLLUX and CASTOR, however, data were immature and, as a result, median OS was not reached in any treatment arm (Table 1).

Significant PFS benefits for study treatment versus control were also reported in all four trials. Median PFS in ASPIRE was 26.3 months with carfilzomib/lenalidomide/dexamethasone versus 17.6 months with lenalidomide/dexamethasone (HR 0.69; 95% CI 0.57, 0.83; *p* < 0.0001) [24, 33], while in ENDEAVOR median PFS was 18.7 months with carfilzomib/dexamethasone versus 9.4 months with bortezomib/dexamethasone (HR 0.53; 95% CI 0.44, 0.65; *p* < 0.0001) [23]. Median PFS was not reached with daratumumab/lenalidomide/dexamethasone in POLLUX and with daratumumab/bortezomib/dexamethasone in CASTOR [34, 37]. Median PFS with lenalidomide/dexamethasone (POLLUX) and daratumumab/bortezomib/dexamethasone (CASTOR) was 17.5 months (HR 0.37; 95% CI 0.28, 0.50; *p* < 0.0001) and 7.1 months (HR 0.33; 95% CI 0.26, 0.43; *p* < 0.0001), respectively.

No major differences in safety outcomes were observed between study treatment and comparator in any of the four trials (Supplemental Table 2) with the exception of ENDEAVOR, in which the risk ratio (95% CI) for time to Grade ≥ 2 peripheral neuropathy was 0.11 (0.07, 0.16) in favor of carfilzomib/dexamethasone versus bortezomib/dexamethasone (*p* < 0.0001). For Grade ≥ 3 peripheral neuropathy, the risk ratio (95% CI) was 0.16 (0.08, 0.31) (*p* < 0.0001).

## Discussion

Patients with MM often experience impaired HRQoL [11–13]. For example, patients enrolled in the ASPIRE and ENDEAVOR studies had EORTC QLQ-C30 global health status/QoL scores of ~ 50–60 of a maximum of 100 [24, 35, 36]. It is therefore important to avoid further deterioration wherever possible. In this analysis, HRQoL deteriorations were observed across all four phase III RRMM trials included here, although indirect, descriptive comparison across these trials suggests that the risk of HRQoL deterioration is less with carfilzomib-based than with daratumumab-based therapy. Results from the phase III ASPIRE and ENDEAVOR trials also show that a carfilzomib-based regimen has benefits over comparator treatments in many QoL domains of the EORTC QLQ-C30, including both functional and symptoms subscales. The benefit was particularly apparent on the global health status scale, where significant reductions of 23% and 21% in the risk of HRQoL deterioration were observed in ASPIRE and ENDEAVOR, respectively. In comparison, in POLLUX and CASTOR, the reductions in risk of global health status/QoL deterioration were 6% with daratumumab/lenalidomide/dexamethasone versus lenalidomide/dexamethasone and 4% with daratumumab/bortezomib/dexamethasone versus bortezomib/dexamethasone, respectively. In terms of the EORTC QLQ-C30 functional subscales, HRQoL benefits were observed for daratumumab-based treatment versus comparator only on social functioning. With respect to the individual symptoms scales, significant benefits in favor of carfilzomib-based therapy over comparators were seen for the constipation subscale in both the ASPIRE and ENDEAVOR trials, and additionally for the nausea/vomiting, insomnia, appetite loss, and diarrhea subscales in the ENDEAVOR trial. In contrast, there were no significant differences on the symptom subscales between daratumumab-based therapy and comparators in the POLLUX and CASTOR trials.

To put the results of this analysis in context with other published HRQoL trials in patients with RRMM, the TOURMALINE-MM1 study of lenalidomide/dexamethasone with or without ixazomib showed no differences in EORTC QLQ-C30 scores after a median of 23 months of follow-up, and no significant difference in OS was observed between treatments [17]. In the MM-003 study of pomalidomide plus low-dose dexamethasone versus high-dose dexamethasone, however, combination therapy was associated with a greater probability of improved HRQoL and prolonged time to HRQoL worsening [19]. The OS benefit in the subgroup of patients with ≥ 2 prior treatments was 5 months. In the PANORAMA-1 study, HRQoL scores after 48 weeks of treatment with bortezomib/dexamethasone, with or without panobinostat, showed no benefit, and no significant difference in OS was observed between treatments [43].

Interestingly, it has been reported that there may be a relationship between improvement in HRQoL and treatment response in patients with cancer [36, 44]. In a sub-analysis of the ENDEAVOR trial, patients receiving carfilzomib/dexamethasone who were classified as responders (i.e., those with a partial response or better) experienced significant improvement on the EORTC QLQ-C30 global health status scale relative to non-responders [45].

All four trials included in the current analysis showed improved median PFS (the primary endpoint in each trial) for carfilzomib- or daratumumab-based therapy versus comparator [23, 24, 28, 29]. Furthermore, carfilzomib was associated with a median prolongation in OS of almost 8 months, regardless of whether it was used in combination with lenalidomide/dexamethasone or dexamethasone alone [5, 8, 23, 24, 28, 29]. Median PFS and OS have not yet been reached for daratumumab in published reports of final analyses, so it is unclear what magnitude of efficacy benefit is associated with this agent.

Tolerability was generally similar across the four trials [23, 24, 28, 29], although there was a significantly lower incidence of peripheral neuropathy symptoms of Grade ≥ 2 with carfilzomib/dexamethasone (6%) versus bortezomib/dexamethasone (32%; *p* < 0.0001) in the ENDEAVOR head-to-head trial [23]. Indeed, bortezomib-induced peripheral neuropathy has a substantial impact on HRQoL and is difficult to manage, usually requiring reduction, interruption, or cessation of therapy [46].

To the best of our knowledge, this is the first descriptive comparison of HRQoL during carfilzomib- and daratumumab-based therapy, and the first time HRQoL has been assessed prospectively in a large cohort of similar patients with RRMM (carfilzomib *n* > 1700; daratumumab *n* > 1000). Another strength of the trials was the very high return rates for QoL questionnaires in a population of severely ill patients, for whom completion of QoL questionnaires may be considered too demanding and/or time consuming. In addition, some patients may have died or relapsed before questionnaires could be returned. There were, however, some limitations to the study. For example, this was an indirect descriptive comparison of carfilzomib- and daratumumab-based therapy, and direct, prospective, head-to-head trials are required to explore any potential differences between these agents in their impact on HRQoL and outcome. Furthermore, as the protocol-defined treatment cycles in which HRQoL was assessed differed between trials (see Table 1), this may have had an impact on the recorded time to deterioration. Any bias introduced in this way is expected to be minor, however, and mitigated by the use of HRs in these analyses. It is possible, however, that more frequent study visits could result in more intensive caregiver–patient interactions, with a positive impact on HRQoL. As these were prospective clinical trials, HRQoL measurements were made only during study treatment and stopped after disease progression or death. Another limitation was the potential for under-reporting of HRQoL impact as a result of different treatment durations between studies. For example, there was a maximum of eight cycles of daratumumab/bortezomib/dexamethasone in the CASTOR study, after which patients received daratumumab monotherapy. There were also other methodological differences between trials, such as different patient numbers; dates of recruitment; patient characteristics; HRQoL instruments and cut-off points used, including protocol-defined MIDs; and pre- and post-study treatments. Moreover, the open-label designs of the four studies meant that patients were aware of their treatment assignment and response, which may have affected their answers to HRQoL questions, although no analysis of potential correlations between tumor responses and HRQoL were conducted as part of the AMNOG assessment.

In conclusion, this analysis has demonstrated the feasibility of HRQoL measurement with high questionnaire return rates in large populations of severely ill patients with RRMM. Currently, carfilzomib-based therapy is the only treatment for RRMM that has demonstrated significant differences versus study comparators in OS, global health status/QoL and other functional and symptom scales. Furthermore, descriptive analysis of the available HRQoL data across trials suggests potential benefits for carfilzomib-based therapy over standard therapy, as well as over daratumumab-based therapy. With the introduction of more effective therapies, the improvements in OS over time in patients with MM mean that the disease is now a chronic disorder, and so patients’ HRQoL should be assessed regularly in prospective MM trials [47]. There may also be a role for QoL monitoring tools and apps, such as CANKADO (https://cankado.com/) to improve the quality of patient care.

## Electronic supplementary material

Below is the link to the electronic supplementary material.

Supplementary material 1 (PDF 154 kb)
