# Supplementary material for: Health-related quality of life of carfilzomib- and daratumumab-based therapies in patients with relapsed/refractory multiple myeloma, based on German benefit assessment data
Source: Qual Life Res. 2019 Sep 24;29(1):69–79. doi: 10.1007/s11136-019-02307-5 (PMC6962286; doi:10.1007/s11136-019-02307-5)
Supplement: Supplementary file 1 — Supplementary material 1 (PDF 154 kb) [file 11136_2019_2307_MOESM1_ESM.pdf]

Supplemental Table 1 Return rates for quality of life questionnaires in patients who were alive and receiving study treatment at each timepoint in the ASPIRE, ENDEAVOR, POLLUX, and CASTOR trials

**ASPIRE**					
**EORTC QLQ-C30**	**Baseline**	**Cycle 3**	**Cycle 6**	**Cycle 12**	**Cycle 18**
KRd, *n/N* (%)	376/394 (95.4)	357/382 (93.5)	327/363 (90.1)	256/305 (83.9)	227/262 (86.6)
Rd, *n/N* (%)	369/394 (93.7)	338/372 (90.9)	284/341 (83.3)	212/263 (80.6)	148/186 (79.6)
**EORTC QLQ-MY20**					
KRd, *n/N* (%)	373/394 (94.7)	353/382 (92.4)	324/363 (89.3)	255/305 (83.6)	223/262 (85.1)
Rd, *n/N* (%)	366/394 (92.9)	334/372 (89.8)	284/341 (83.3)	211/263 (80.2)	147/186 (79.0)
**ENDEAVOR**					
**EORTC QLQ-C30, EORTC QLQ-MY20, FACT/GOG-Ntx**	**Baseline**	**Cycle 3**	**Cycle 6**	**Cycle 12**	**Cycle 18**
Kd, *n/N* (%)	407/464 (87.7)	383/408 (93.9)	298/343 (86.9)	137/159 (86.2)	41/44 (93.2)
Vd, *n/N* (%)	392/465 (84.3)	336/388 (86.6)	222/254 (87.4)	73/80 (91.3)	11/12 (91.7)
**POLLUX**					
**EORTC QLQ-C30**	**Baseline**	**Cycle 3**	**Cycle 6**	**Cycle 12**	**Cycle 18**
DRd, *n/N* (%)	252/286 (88.1)	234/269 (87.0)	229/254 (90.2)	197/216 (91.2)	36/39 (92.3)
Rd, *n/N* (%)	259/283 (91.5)	212/263 (80.6)	194/225 (86.2)	143/165 (86.7)	14/20 (70.0)
**CASTOR**					
**EORTC QLQ-C30**	**Baseline**	**Cycle 3**	**Cycle 6**	**Cycle 12[a]**	**Cycle 18[a]**
DVd, *n/N* (%)	226/251 (90.0)	197/218 (90.4)	192/207 (92.8)	71/77 (92.2)	1/2 (50.0)
Vd, *n/N* (%)	219/247 (88.7)	187/209 (89.5)	151/165 (91.5)	–	–

[a]Vd therapy was discontinued after eight cycles in both treatment arms; in the DVd arm, daratumumab monotherapy continued until cycle 18

DRd daratumumab/lenalidomide/dexamethasone, *DVd* daratumumab/bortezomib/dexamethasone, *EORTC* European Organisation for Research and Treatment of Cancer, *FACT/GOG-Ntx* Functional Assessment of Cancer Therapy/Gynecologic Oncology Group-Neurotoxicity scale, *Kd* carfilzomib/dexamethasone, *KRd* carfilzomib/lenalidomide/dexamethasone, *MY20* EORTC 20-item Myeloma Specific Questionnaire, *QLQ-C30* EORTC 30-item Quality of Life Questionnaire, *Rd* lenalidomide/dexamethasone, *Vd* bortezomib/dexamethasone

Supplemental Table 2 Time to adverse events with carfilzomib-based therapy and daratumumab-based therapy versus comparator

**Carfilzomib-based therapy**	**ASPIRE**				**ENDEAVOR**			
	**KRd (*n* = 396)**	**Rd (*n* = 396)**	**HR (95% CI)**	***p* value**	**Kd (*n* = 464)**	**Vd (*n* = 465)**	**HR (95% CI)**	***p* value**
Any AE	0.3 (0.1, 0.3)	0.4 (0.3, 0.5)	– (–)	–	0.1 (0.1, 0.2)	0.2 (0.2, 0.3)	– (–)	–
Serious AE	12.7 (0.1, 16.0)	15.4 (12.7, 19.1)	1.06 (0.89, 1.27)	0.151	10.9 (8.8, 14.3)	16.4 (13.8, 22.7)	1.22 (1.01, 1.47)	0.040
Severe AE (Grade ≥3)	16.9 (13.7, 22.7)	20.9 (18.7, 27.3)	1.11 (0.92, 1.34)	0.290	3.1 (2.3, 4.0)	2.9 (2.4, 3.8)	1.06 (0.92, 1.24)	0.413
Discontinuation due to AE								
Total study medication	– (–)	– (–)	0.93[a] (0.70, 1.23)	0.683	33.1 (23.1, –)	– (33.9, –)	1.11[a] (0.90, 1.37)	0.530
≥ 1 study medication	– (–)	– (–)	1.11[a] (0.90, 1.37)	0.370	– (31.7, –)	– (35.5, –)	1.15[a] (0.91, 1.46)	0.248
**Daratumumab-based therapy**	**POLLUX**				**CASTOR**			
	**DRd (*n* = 286)**	**Rd (*n* = 283)**	**HR (95% CI)**	***p* value**	**DVd (*n* = 251)**	**Vd (*n* = 247)**	**HR (95% CI)**	***p* value**
Any AE	–	–	–	–	–	–	–	–
Serious AE	14.3 (–)	16.8 (–)	1.14 (0.90, 1, 44)	0.290	14.1 (–)	– (–)	1.24 (0.92, 1.65)	0.153
Severe AE (Grade ≥ 3)	1.0 (–)	3.4 (–)	1.39 (1.15, 1.68)	< 0.001	1.2 (–)	1.9 (–)	1.42 (1.14, 1.77)	0.002
Discontinuation due to AE								
Total study medication	– (–)	– (–)	0.99[a] (0.58, 1.71)	< 0.999	– (–)	– (–)	0.98[a] (0.56, 1.71)	< 0.999
≥ 1 study medication	– (–)	– (–)	– (–)	– (–)	– (–)	– (–)	1.00 (0.67, 1.50)	< 0.999

[a]Risk ratio
Data are presented as median (95% CI) months, with no adjustment for treatment exposure
AE adverse event, *CI* confidence interval, *DRd* daratumumab/lenalidomide/dexamethasone, *DVd* daratumumab/bortezomib/dexamethasone, *HR* hazard ratio, *Kd* carfilzomib/dexamethasone, *KRd* carfilzomib/lenalidomide/dexamethasone, *Rd* lenalidomide/dexamethasone, *Vd* bortezomib/dexamethasone

Supplemental Fig. 1 Forest plot showing hazard ratios for differences between bortezomib/dexamethasone and carfilzomib/dexamethasone (ENDEAVOR trial) for time to ≥ 10-point deterioration in subscales of the EORTC QLQ-MY20

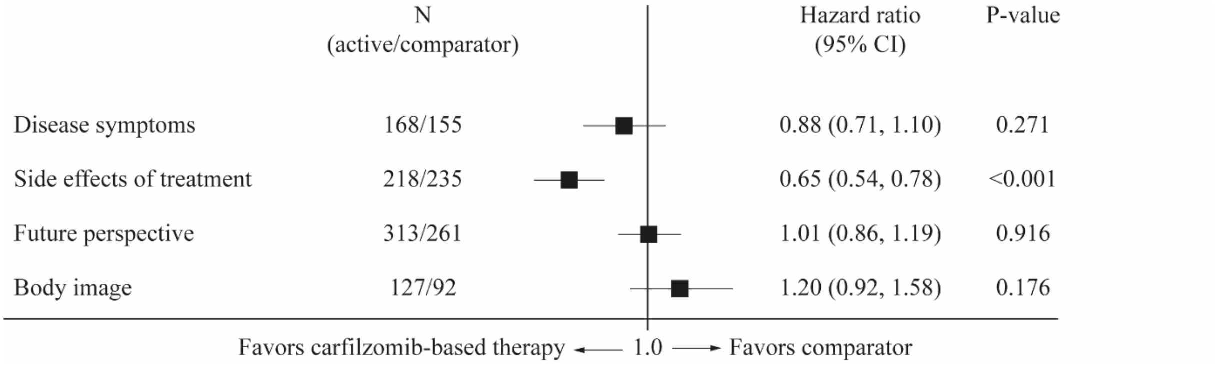

CI confidence interval, *EORTC* European Organisation for Research and Treatment of Cancer, *QLQ-MY20* EORTC 20-item Myeloma Specific Questionnaire
